# Perceptions of Barriers and Facilitators to a Pilot Implementation of an Algorithm-Supported Care Navigation Model of Care: A Qualitative Study

**DOI:** 10.3390/healthcare11233011

**Published:** 2023-11-21

**Authors:** Rebecca K. Pang, Nadine E. Andrew, Velandai Srikanth, Carolina D. Weller, David A. Snowdon

**Affiliations:** 1 Department of Medicine, Peninsula Clinical School, Central Clinical School, Monash University, Frankston, VIC 3199, Australia; rebecca.pang@monash.edu (R.K.P.); velandai.srikanth@monash.edu (V.S.); david.snowdon@monash.edu (D.A.S.); 2 Professorial Academic Unit, Peninsula Health, Frankston, VIC 3199, Australia; 3 National Centre for Healthy Ageing, Frankston, VIC 3199, Australia; 4 School of Nursing and Midwifery, Monash University, Clayton, VIC 3800, Australia; carolina.weller@monash.edu

**Keywords:** care navigation, risk algorithm, staff perception, i-PARIHS, hospital readmission

## Abstract

We aimed to explore managerial and project staff perceptions of the pilot implementation of an algorithm-supported care navigation model, targeting people at risk of hospital readmission. The pilot was implemented from May to November 2017 at a Victorian health service (Australia) and provided to sixty-five patients discharged from the hospital to the community. All managers and the single clinician involved participated in a semi-structured interview. Participants (*n* = 6) were asked about their perceptions of the service design and the enablers and barriers to implementation. Interviews were transcribed verbatim and analysed according to a framework approach, using inductive and deductive techniques. Constructed themes included the following: an algorithm alone is not enough, the health service culture, leadership, resources and the perceived patient experience. Participants felt that having an algorithm to target those considered most likely to benefit was helpful but not enough on its own without addressing other contextual factors, such as the health service’s capacity to support a large-scale implementation. Deductively mapping themes to the integrated Promoting Action on Research Implementation in Health Services (i-PARIHS) framework highlighted that a formal facilitation would be essential for future sustainable implementations. The systematic identification of barriers and enablers elicited critical information for broader implementations of algorithm-supported models of care.

## 1. Introduction

Over the past decades, health services have trialled numerous models of care to specifically target frequent users of hospital services in an attempt to reduce hospital readmissions [1]. Some models are hospital-based and focus on improving hospital discharge processes [2,3], whereas others are community-based to ensure a continuum of patient care [4,5]. Care navigation is one such model that has shown some promise in bridging the gap between hospitals and the community [6]. It is typically characterised by the use of a navigator, usually a health professional, to support the patient transition and coordinate their care between the hospital and community [7,8]. The care navigator is responsible for coordinating care in the community for a patient and is an advocate for their health and social care needs, but they are not directly involved in delivering clinical care [9]. For example, they may assist with arranging appointments and transportation to medical clinics and relevant community services in order to promote self-care and person-centred care in chronic disease management.

Care navigation is a resource-intensive intervention that can impose considerable additional costs to healthcare providers [10]. These costs need to be offset by a sufficiently large reduction in hospital readmissions for the intervention to be cost-effective from a provider perspective. Algorithms embedded within electronic health record systems are an attractive mechanism for improving the targeting of these care types with potential to ensure that care is directed towards those most likely to benefit. Whilst a number of studies have used hospital data to identify specific conditions responsible for hospital admissions for targeted support [11,12], there are a growing number of studies where algorithms derived within electronic health record systems have been used to identify patients most at risk of hospital readmission [10,13,14]. While two of these studies showed the algorithm-supported care navigation to be effective [10,13], another found it to be no more effective than the usual care [14]. These differences can be attributed in part to heterogeneity in the components of the intervention; methodological flaws, such as small sample sizes or quasi-experimental study designs; and implementation challenges, including a poor uptake by participants or organisations with poor compliance by participants and/or fidelity in intervention delivery [10,14].

Despite the potential for algorithm-supported care navigation to reduce hospital readmissions in those most at risk, there is limited information available to guide the implementation of this type of care. Of these three studies, only one by Brown et al. [10] was accompanied by a qualitative evaluation of the trial to obtain the perspectives of the case managers providing the intervention [15]. Obtaining feedback on organisational barriers and enablers to implementing algorithm-supported care navigation at the managerial level is critical for sustainable implementation within health services. However, to our knowledge, the perception of the managers responsible for maintaining and championing these types of organisation-level interventions has not been previously explored.

The “integrated Promoting Action on Research Implementation in Health Services” (i-PARIHS) framework [16,17,18] is a theoretical implementation framework, which has been used extensively in research to (1) explain how barriers and enablers influence implementation outcomes (e.g., health care professional behaviour) and (2) guide the implementation of healthcare innovations [19,20]. The i-PARIHS framework consists of four main constructs; these include innovation, recipients, context and facilitation. In the context construct, it is further subdivided into three layers (local, organisational and external) to show the complex interaction of implementation science. The i-PARIHS framework was informed by Roger’s Diffusion of Innovations [19,21], which describes how an innovation’s pros and cons, the characteristics of adopters (i.e., recipients), opinion leaders’ social influence, and the larger social and political context can influence the diffusion, or uptake, of innovations. Drawing on this theory, the i-PARIHS framework highlights the complexities and multiple contextual layers involved in health service implementations, where ongoing monitoring; the level of facilitation, support and adaptation by health services; and collaboration with external agents, such as the government or private sectors, are required [22]. Interpreting staff perceptions of the barriers and enablers to implementing algorithm-supported care navigation through the lens of the i-PARIHS framework would facilitate a deep understanding of implementation and contextual factors that could guide the future implementation and scale-up of this complex model of care within electronic clinical systems. 

The aim of our study was to explore managerial and project staff perceptions of a pilot care navigation model targeting people at risk of hospital readmission, identified using an electronic health record-derived readmission risk algorithm using qualitative evaluation methods. 

## 2. Materials and Methods

### 2.1. Design

We conducted a qualitative descriptive study following a framework approach, using inductive and deductive techniques [23]. In-depth semi-structured interviews were conducted to explore participant perceptions of the model of care. During interviews, we explored participant perceptions of the use of the risk prediction algorithm to identify appropriate participants for the service; the enablers and barriers to the implementation of this model of care; the perceived impact on hospital readmission and the quality of care; and the feasibility of implementing this model across the health service in a sustainable manner. 

Ethics approval was obtained for the study from the participating health service’s Health Human Research and Ethics Committee (approval no. HREC/LNR/48747/PH-2019-162719(v2)).

### 2.2. Setting and Model of Care 

Targeted care navigation was piloted from May 2017 to November 2017 at a single Victorian health service (Australia). Eligible patients were identified through a hospital readmission risk algorithm provided to hospitals across the state by the Victorian Department of Health [24] as part of the HealthLinks Chronic Care (HLCC) state government project. The algorithm score was based on acute admissions in the past six months, emergency department visits in the previous three months, patient demographics and various chronic health conditions, the exact details of which can be found in our quantitative evaluation of the pilot study [25]. The care navigation was delivered post-discharge by an experienced clinician, who was a registered nurse and was not directly involved in delivering clinical care either during the admission or post-discharge. The care navigation consisted of a weekly phone call with additional follow-up calls as required for a month [25]. 

### 2.3. Participants

All staff (*n* = 6) involved in the pilot implementation from the executive level down to the project manager/team leader level plus the single clinician who delivered the care navigation were eligible to participate in the semi-structured interview. All were invited via email by the researcher, to elicit their interest in participating. A phone call was used as follow-up if the researcher did not receive a reply two weeks after sending the email. All participants provided written informed consent prior to participating in the interview. 

### 2.4. Data Collection

Face-to-face interviews were conducted by two experienced research coordinators with backgrounds in health administration and allied health using a semi-structured interview guide between June and August 2018. Interview questions were developed with reference to the Theoretical Domains Framework (TDF) [26] to ensure comprehensive information was obtained to inform the future implementation and scale-up of the project. The interview schedule for the participant interviews explored the useability, practicality, sustainability, benefits (personal, professional and organisational) and perceived enablers/barriers of the targeted care navigation model (Appendix A). Neither of the interviewers were involved in the planning or implementation of the pilot study. Both had prior experience interviewing clinicians, allowing us to achieve credibility whilst maintaining sufficient independence to minimise the possibility of social desirability bias [27]. Both were briefed about the research topic prior to conducting interviews. Interviews were approximately 60 min and were conducted in a private room at the health service, during business hours. The interviews were audio-recorded and professionally transcribed. A copy of the transcript was provided to all participants, who were encouraged to make amendments to the transcripts if they believed that the transcript did not accurately represent what they intended to say [28]. No changes or feedback were made by them. 

### 2.5. Data Analysis

Data were analysed according to Ritchie and Spencer’s [23] five-stage framework analysis, using both inductive and deductive techniques. A combination of inductive and deductive techniques was used to ensure that all implementation determinants (i.e., enablers and barriers) were identified. Specifically, deductively mapping to the iPARIHS framework provided a structured, and reproducible, method for identifying implementation determinants [19,29]. Inductive analysis ensured that determinants that may not be captured by the iPARIHS framework were not overlooked and the nuance and context were not lost [30,31]. 

The first stage of inductive analysis (familiarisation) involved all six interview transcripts being read and analysed by two team members each, using an inductive approach, with three authors analysing four transcripts. Step two (identifying a thematic framework) involved two of the authors developing an initial coding framework based on the preliminary analysis performed in step one. This detailed the codes, their descriptions and example quotes. All participants reviewed this coding framework and agreed on the final version following discussion based on multiple iterations. Step three (indexing) involved the same two authors coding all six transcripts in NVivo using the established coding framework [32]. They met two times to cross-check the coding of transcripts and discuss any points of ambiguity with a third author. The coding framework was modified as required during the index process. The fourth stage (charting) involved identifying patterns in the data, refining and confirming themes from the developed codes in step three. The fifth and final stage (mapping and interpretation) involved interpreting the data in light of the existing care navigation and implementation research literature. 

Themes and codes were deductively mapped to the i-PARIHS framework [16,17,18]. The i-PARIHS framework was chosen as it draws on the TDF (used to develop the interview questions) as a theoretical concept and was developed to assist with the complexities of complex multidisciplinary interventions that involved multiple contexts, in this case, government, health service and program levels [17]. The themes and codes created during our inductive analysis were first mapped to the i-PARIHS framework by two authors then refined and confirmed with the third author.

Our sample of six participants includes all staff who were involved in the pilot implementation of the care navigation and meets the recommended number of participants for thematic analysis of small qualitative projects [33].

## 3. Results

All eligible staff who were invited participated in the study. Of the five organisational managers, three were allied health professionals (physiotherapy, occupational therapy, social work) and two were medical professionals (specialist physician, general practice/primary care). All participants had worked in their roles for at least five years with the same health service. The clinician who delivered the care navigation was a nurse and had been working for more than twenty years across all sectors within the health service. 

### 3.1. Themes and Subthemes

One overarching theme and four subthemes were constructed from the qualitative data (Table 1). The overarching theme was “an algorithm alone is not enough” to support the implementation of a care navigation model. The four subthemes described the main factors that influenced participants’ perspectives on the implementation of the model of care: the health service culture, leadership, staff and resources, and the patient experience of care.

#### 3.1.1. An Algorithm Alone Is Not Enough

Overall, participants felt that the model of care improved the quality of care provided to patients. They could see value in having a risk algorithm to support the model of care and believed the algorithm provided a useful starting point for identifying patients most at risk of readmission. The algorithm was thought to assist in directing limited resources to people who needed it most:

*“One challenge is identifying the patients who are vulnerable and likely to represent to hospital…an algorithm is a really good start as this helps target the right people and they get the right support. I don’t think the algorithm alone is enough’ but it is a good start.”*

*(P1)*

*“With high scores there was definite a chance of representing. It’s not perfect but it’s better than just going by who presented three times in the last year. Another way of weeding out the patients we needed to see compared to the patients that just needed a general practitioner visit.”*

*(P3)*

Although participants identified that the risk algorithm helped to identify patients at risk of readmission, participants expressed that the risk algorithm alone was not enough to support the care navigation model. They reported that the health service lacked the capacity and capability to support the implementation of the model of care, citing difficulty in fully engaging different stakeholders across the health system and coordinating system-wide changes:

*“If you want to look at how you can reduce the impact these patients have on acute services—expensive bed-based service—it (the algorithm) does capture patients from this perspective. It’s like early intervention in chronic disease program, and there’s a whole body of work that needs to happen before people get to that point.”*

*(P4)*

*“This project was not just about implanting a new model of care in a specific area; this is broad change at a significant level that is going to affect not just one unit of medicine but multiple and this is a hard and difficult thing to coordinate.”*

*(P4)*

Targeted care navigation was seen as a community-based model of care, a significant shift from traditional hospital-based models. The significance of this change was recognised by participants’ beliefs that it may take several years before health services are ready to successfully implement these types of models of care:

*“It’s years away as it’s a significant change and would take at least ten years for it to mature enough. Considering we have talked about it for three and a half years and only done a small pilot project. So, given that’s our baseline for how long implementation takes to occur it will take a lot of change to support it.”*

*(P4)*

#### 3.1.2. Health Service Culture

Participants reported that implementing the targeted care navigation model was challenging in an environment that favoured traditional medicalised models of care over holistic multidisciplinary care:

*“We are still adopting specialist care largely focused on single diseases, and with training programs that do not have any involvement or minimal training in community health. Concerns about control of patient care being lost if multidisciplinary care plans are adopted.”*

*(P3)*

Participants perceived that this culture could lead to organisational hesitancy in shifting the provision of care from the hospital to the community, presenting a barrier to the implementation of care navigation:

*“Another one would be the culture and capacity to change. Culturally the organisation was invested in bed-based care and hesitant to pilot innovative projects with a culture of learning around working with these complex individuals.”*

*(P1)*

The participants reported conflict between their professional identity and the current health care culture. They expressed a preference for community-based holistic care and believed that the aims of targeted care navigation aligned well with their professional values:

*“It (targeted care navigation) sits strongly with me and my professional identity…my profession is about enabling people to better manage their own health and wellbeing.”*

*(P1)*

*“Given my background is predominantly in primary care I’m passionate about primary care and providing care and services outside of hospital and in the community.”*

*(P2)*

#### 3.1.3. Leadership

Participants reported a need for stronger leadership and direction from the organisation for effective implementation. They reported opaque communication between the government and the health service, leading to a lack of clarity on the ground about funding and how to apply the risk algorithm:

*“We needed higher engagement from the department of health with our health service, and performance meetings and looking at engaging them on this journey first.”*

*(P3)*

*“They didn’t give us clarity early on of what the readmission risk score actually meant…you’d have to think there was an increase in risk as your score went up.”*

*(P3)*

Participants also reported a lack of incentive to increase primary and community health service activity based on current funding models. They reported a need for the government to provide financial incentives for reducing hospital admissions and increasing community healthcare activity:

*“I think at that high level the direction given by the government about the funding model was not clear.”*

*(P4)*

*“The hospital can think this is a loss if you move patients away from current hospital-based funding.”*

*(P3)*

In the context of current funding models, participants believed that the organisation perceived a financial risk associated with the project, which restricted the scope of the pilot project to a limited number of patients:

*“There was a perspective that there was a lot of risk with the project, so we didn’t get support to be on board with the project in its entirety.”*

*(P2)*

*“At the time when we were involved there wasn’t a great appetite for risk and so we were tasked with providing a very limited level of service. We kind of put a toe in the water and did something, so we were involved but not do a whole lot.”*

*(P5)*

Participants reported that this reduced prioritisation made it difficult to coordinate care across the health service’s hospital and community care settings:

*“It’s difficult—it’s a system change across the health service and it’s near impossible if you don’t have strong organisational support.”*

*(P2)*

Participants were also unsure of the key performance indicators imposed by the government to demonstrate the project’s success. This led to confusion about the parameters for success despite an awareness of the overall aim of the project:

*“The aim was to prevent hospital readmissions but key performance indicators were not clarified.”*

*(P6)*

Despite reservations towards supporting a comprehensive model, participants with managerial roles reported an organisational commitment to the pilot project with an intent to trial a larger implementation again in the near future. However, they stipulated that lessons from the current project would need to be incorporated. These lessons included increased funding and improved coordination of healthcare across the health service:

*“It’s critical that we do implement a (targeted care navigation) model. Looking at how we evaluate our out of hospital services and at how we can better care for patients outside of hospital walls. I think a (targeted care navigation) model will be a driver to do this.”*

*(P1)*

#### 3.1.4. Staffing and Resources

Participants reported that the primary enabler of the targeted care navigation model was the sole clinician who delivered the care. They emphasised the importance of having a role dedicated to providing care navigation and a clinician in that role who could strongly advocate for patients’ needs. However, participants were unsure if the perceived improvement in the quality of care was reflective of the model or the clinician:

*“The other big enabler was the clinician. Because of their personality they got a lot more done than someone who was meeker or milder and not pushy for the patient… It was never clear to me, if we were to get positive results, how much of it would be process or person related.”*

*(P5)*

However, there was also acknowledgement that there were not enough clinical staff dedicated to the project, which led to difficulty coordinating care for patients and a limited number of eligible patients receiving care navigation:

*“(The project was negatively) Impacted by only having one part-time staff member. The more people you have, the more you can facilitate the project.”*

*(P6)*

*“A whole group of patients may have missed out because (of) resourcing due to it only being a pilot.”*

*(P4)*

Participants also reported that the clinician who provided care navigation held a dual role: first to deliver the care navigation intervention to patients and also to facilitate the implementation by promoting the model of care within the hospital, identifying problems and finding solutions to encourage uptake. They felt that this aspect would need to be enhanced and better supported were the model to be scaled up:

*“The clinician did an amazing job advocating for patients to be seen before they left hospital.”*

*(P4)*

*“In an ideal world we wouldn’t have just one person to provide care navigation and facilitate this.”*

*(P4)*

Although participants believed there was value in using a risk algorithm to identify and target those most likely to benefit from care navigation, they also reported that the information technology (IT) systems at the time did not enable high-risk patients to be automatically “flagged” in “real time”. Instead, the clinician coordinating the targeted care navigation needed to rely on a daily printed spreadsheet. This made the identification of high-risk patients and coordination of their care less efficient:

*“You need good, sophisticated IT systems to flag patients at risk.”*

*(P2)*

Participants were confident that the community health services required to support patients who were discharged from the hospital were already in existence and that there was no need to develop new services:

*“We have programs to build upon and some services can already be used without reinventing the wheel from scratch and building a whole new program. We don’t want to create another silo of care for people.”*

*(P4)*

However, participants recognised that the current systems were not good at facilitating communication between clinical services or seamlessly coordinating care for people discharging from hospital (e.g., referral processes). They identified this as a barrier to targeted care navigation, as the clinician was required to work within these systems to coordinate appropriate care:

*“Our clinical systems are quite complex and don’t necessarily facilitate good connected care—we need systems to do that.”*

*(P1)*

Participants also reported that the model itself needed to be more agile and responsive to the needs of the patient, rather than being a fixed-term intervention:

*“You have patients that have social complexities that will always be at risk and they need more intervention than a four-week program.”*

*(P2)*

#### 3.1.5. Patient Experience of Care

There was a strong consensus that targeted care navigation improved patients’ experience of care. They believed that the follow-up phone calls and support following discharge were likely a source of great reassurance for patients:

*“I think the patients that were involved really appreciated having the follow-through, from the organisation, with their care. They felt they were cared about with the follow-up phone calls and sometimes it’s the simple things and it doesn’t need to be complicated.”*

*(P4)*

Participants also reported that they felt the model of care reassured clinicians on the hospital wards that the patients in the project would be cared for following discharge:

*“Staff on the wards really felt comfortable that someone would follow them (patients) up.”*

*(P3)*

However, participants were unsure if the improvement in experience led to better outcomes. This led them to question the importance of an improvement in patient experience without an obvious reduction in hospital readmissions:

*“As nice as it was for patients for feeling better, the readmission rate wasn’t reduced. The quality of care improved, the quality of discharge improved but in terms of the outcome we were looking at there was no obvious benefit.”*

*(P5)*

Participants also believed that improving the quality of care would have little impact on some people with chronic diseases who are non-compliant with recommendations and comfortable with hospital-based care:

*“Some patients don’t do what you think they should do, which is an issue.”*

*(P4)*

*“People with chronic illness who are probably just expected to be readmitted. These people actually do not mind being in hospital, and coming back to hospital was not such a bad thing.”*

*(P6)*

### 3.2. Mapping Themes to i-PARIHS Framework

**Figure 1:**
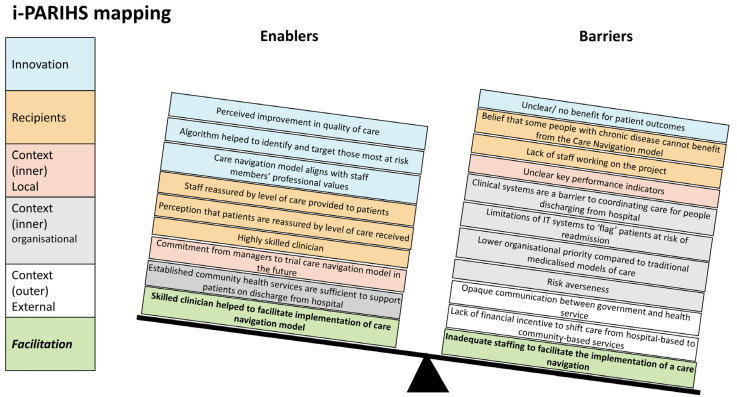
Mapping of themes of targeted care navigation implementation to i-PARIHS framework presented in a weighing scale form.

In mapping the themes to the i-PARIHS framework, we identified findings of relevance to three of the four constructs of the i-PARIHS framework: innovation, recipients and context (Table 2). The majority of challenges fell under the context construct, and the imbalance between enablers and barriers negatively increased as the context level moved outward, i.e., from the local level to the health system level (Figure 1). It also showed that the innovation (targeted care navigation) was largely supported and the participants were mostly positive about their ability to implement it if they had more resources. In the facilitation construct, we found that more resources and a dedicated facilitator role, other than the clinician delivering the care navigation, would be required to successfully implement the model of care across the health service.

## 4. Discussion

We used qualitative methods to explore the managerial and project staff perceptions of the implementation of an algorithm-supported care navigation project targeting people at risk of hospital readmissions. Findings suggested that the model of care was well accepted by the project team within the health service and aligned with the culture of the team. However, the use of an algorithm to identify and target those most at risk of hospital readmission was not enough on its own to support a successful implementation, which was considered to be difficult within the broader context of the health service and health system at the time. Mapping themes to the i-PARIHS enabled us to identify elements at the local program, health organisation and state health department levels that could enhance the future implementation of such programs. The inability to map themes and subthemes to the *facilitation* construct in the i-PARIHS framework highlighted a potential limitation that would need to be addressed for future service-wide implementation. 

Overwhelmingly, interviewees’ perceptions of the care navigation project were positive and supported by clinical experience and perceptions of consumer preferences. Targeted care navigation aligned with interviewees’ professional values, which was reflected in their belief that patients experienced a greater quality of care as a result of the program. This belief resulted in a strong commitment and motivation from the management team to trial targeted care navigation and was seen by some as a first step in promoting broader contextual and system changes to enhance patient-centred and integrated healthcare. All members of the project team showed a clear understanding of the model of care, which is essential for future replications or expansions of the model [11,34,35]. The perception that targeted care navigation supported by the algorithm had a clear “relative advantage” over existing practices with regards to the quality of care, despite a belief that the pilot project did not meet its objective of significantly reducing hospital readmissions, also supports the potential for a broader implementation [12,25]. Interestingly, the belief that the model of care did not reduce readmissions was contrary to the results from the quantitative evaluation of the project [25].

In recent years, there has been a focus on developing algorithms imbedded in electronic health records to inform clinical decision making, diagnosis and prognosis. Despite advancements in the development of digital health tools, such as machine learning-supported algorithms, there is limited real-world evidence of how these algorithms can be successfully implemented into clinical practice to facilitate improvements in service delivery and patient outcomes [36,37,38]. Recent studies that have used an algorithm to identify high-risk patients for care navigation have reported a poor uptake by “at risk” patients with 37–40% declining the intervention [10,13] or high cross-over rates to usual care of 75% [14]. Our findings may explain this lack of uptake by highlighting that an algorithm, implemented without appropriate consideration of the broader contexts and systems within which it will be used, is insufficient. Supported by the theory underpinning the i-PARIHS framework, it is clear that the performance of the algorithm (i.e., innovation) is only one construct that must be considered to facilitate its application in healthcare settings. One must also consider how the algorithm integrates with clinicians who use the algorithm (i.e., recipients), patients who receive care that is informed by the algorithm (i.e., recipients) and the healthcare setting within which the algorithm is being used (i.e., context) [17]. Our study has shifted focus from just having an algorithm to understanding how algorithms such as this can be better integrated into clinical systems and workflows to support existing practices and facilitate large scale applications in healthcare settings.

Leadership and the capacity to implement targeted care navigation and the role that health service culture played in supporting these was evident across multiple aspects of the i-PARIHS *context* constructs (local, organisational and government). Participants in our study believed that the health service already possessed some capacity to successfully implement targeted care navigation, such as skilled staff and services that can support people discharging from hospital. However, they also reported that the health service lacked the information technology and clinical systems required to maximise the utility of the algorithm in coordinating these services for seamless care. There was also a perceived hesitancy by some staff to shift certain types of care from the hospital to the community. These findings highlight aspects of the health service that could be further examined, using validated tools, such as the Organizational Capacity for Change Measurement Tool [39], to better understand the organisation’s capacity to implement large-scale change. Although they are outside the scope of this current study, future research should consider such evaluations to inform strategies aimed at improving capacity for change.

Barriers to implementation included the competing priorities of the health service, hesitancy to change, inconsistent communication and unclear expectations. Evidence from the literature suggests that for the implementation to be successful it must fulfil the sub-elements of the organisational context and the organisation also needs to provide good leadership, access to resources and financial incentives. In addition, commitment, coordination and community involvement are required to ensure sustainability [40,41,42,43,44]. Another commonly identified implementation challenge was the perceived ambiguity at the government level, including poorly defined objectives, ill-defined funding models and unclear performance indicators. These types of factors at multiple levels of the healthcare system have been shown to influence the success or failure of implementations [45]. Each level must not only achieve their goals but must also recognise the imperative of joining with other levels to optimise the implementation of the system as a whole [45]. Where goals are in conflict or are not clearly communicated between levels, implementation failure can occur. Importantly, context needs to be considered at all levels to ensure that changes in any level of the system will not impact on the whole project’s sustainability. 

Retrospective mapping of the i-PAHRIS framework and other frameworks for implementation evaluation has been shown to be useful for understanding specific aspects that may have promoted a successful implementation and for identifying areas for improvement that may have been overlooked using inductive analysis on its own [46]. This may be particularly relevant for pilot studies such as ours that are performed to inform an implementation framework for future larger studies that require a strong implementation focus. The “*facilitation*” construct is an important aspect of the i-PARIHS framework required to link all of the constructs together to make the implementation both successful and sustainable [16]. Facilitation is defined as a technique that supports the implementation process by working across professional and organisational boundaries [47,48]. The essential attributes of a facilitator in our context includes strong interpersonal skills, a good understanding of patient needs and services availability, good communication with management and the ability to engage with patients to facilitate change [17,48]. Our inductive analysis identified that the managers acknowledged that the clinician delivering the targeted care navigation had these attributes. Mapping this finding to the iPARIHS framework highlights that the clinician, in the absence of a formally appointed facilitator, undertook a dual role delivering the intervention and facilitating its implementation within the local context. While participants viewed this dual role as problematic, it is likely that the clinicians’ capacity to facilitate change in the local context was enhanced by their understanding of the intervention and experience working within the health service. 

It has been hypothesised that the facilitator role requires a greater level of experience and skill to influence the implementation at the organisational and outer context compared to the local context [17]. Our findings support this hypothesis, as participants identified that, in the absence of an experienced facilitator, there were many barriers that the clinician was unable to address within the organisational and outer context. While not explicitly stated by participants, it is likely that the novice clinician facilitator lacked the skill, support and seniority required to address these complex barriers to implementation [49]. An experienced or expert facilitator would have the ability to play a greater role in addressing the more challenging contextual factors [17], such as communicating the project across the different contextual levels and addressing some of the communication barriers identified in our study. An experienced facilitator would also have the ability to identify and advocate for the systems and resources needed to support the program, working directly with the government and the local organisation. Having experienced facilitators with knowledge of relevant policies and pathways to support the service delivery across the different organisational contexts [50] will be critical for the future expansion of the piloted targeted care navigation project.

A limitation of our study was the small number of participants interviewed. However, the sample size of six represented all clinical and managerial staff involved in the pilot study and provided sufficient information power to achieve our research aims. The rich one-on-one interview dialogue and robust analysis strategy interpreted within an already established theory, i.e., the i-PARIHS frame-work [51], provided sufficiently rich data to inform a broader implementation and scale-up of the targeted care navigation model of care. The limited scope of our sample also meant that we could not include the perspectives of stakeholders external to the health service (i.e., Department of Health). Given that participants identified barriers to implementation that were external to the health service, including opaque communication with the government and a lack of financial incentive to shift care from the hospital to the community, future collaborations with governments will assist in the development of strategies to overcome these barriers. Caution should also be applied in interpreting our results as they may be biased by the majority of participants working at a managerial rather than service delivery level.

The interviews in this study were all conducted by two independent researchers who had no prior involvement in the project. Although this is a strength of the study, as there was likely to be little influence of confirmation bias or social desirability bias [27], the interviewers’ lack of prior experience with targeted care navigation may have led to limited opportunities to challenge participants or explore inconsistencies in relation to stressors that participants associated with the project that were unrelated to the implementation. As this was a retrospective evaluation commissioned well after the completion of the study, it was not possible to interview patients. If the project were to be implemented on a larger scale, patient follow-up would be an important part of the prospective evaluation.

## 5. Conclusions

Using a theory-based approach, we ensured a systematic and comprehensive identification of relevant barriers to and enablers to the pilot implementation of an algorithm-supported targeted care navigation project within a health service. By interpreting our results within the i-PARIHS framework, we have provided a structured evaluation of this pilot trial, allowing for a deep understanding of the implementation and contextual factors that should be considered when implementing complex models of care within electronic clinical systems. In addition, we have contributed new knowledge to the growing field of algorithm-supported models of care, where the focus has traditionally been on their technical implementation rather than how they are operationalised within clinical practice. 

## Figures and Tables

**Table 1:** Summary of theme and subthemes.

Theme/Subthemes	Description	Exemplar Quotes
An algorithm alone is not enough (overarching theme)	Participants believed that the algorithm assisted in directing limited resources to people who needed care. However, they also recognised that the algorithm alone was not enough to support the care navigation model. Targeted care navigation was seen as a community-based model of care and a significant shift from traditional hospital-based models of care. As such, they reported that the successful implementation of targeted care navigation would require substantial resources and leadership from the health service.	“One challenge is identifying the patients who are vulnerable and likely to represent to hospital…an algorithm is a really good start as this helps target the right people and they get the right support. I don’t think the algorithm alone is enough’ but it is a good start.”
Health service culture (subtheme)	Participants reported that implementing the targeted care navigation model was challenging because the health service favoured traditional medicalised models of care over holistic multidisciplinary care. Participants reported conflict with this culture, preferring community-based holistic care to hospital-based medicalised care.	“Another one would be the culture and capacity to change. Culturally the organisation was invested in bed-based care and hesitant to pilot innovative projects with a culture of learning around working with these complex individuals.”
Leadership (subtheme)	Participants reported that opaque communication between the government and health service and a lack of financial incentive to shift care from the hospital to the community negatively impacted the implementation of the targeted care navigation model.	“We needed higher engagement from the department of health with our health service, and performance meetings and looking at engaging them on this journey first.”
Staffing and resources (subtheme)	Participants identified that a key enabler of the targeted care navigation model was the clinician who delivered the care. However, they identified that more clinical staff would ensure that the targeted care navigation model could reach more patients. Participants were also confident that the community health services that existed within the organisation could support people discharging from hospital but identified that better systems were required to facilitate communication and a seamless co-ordination of care between these services.	“In an ideal world we wouldn’t have just one person to provide care navigation and facilitate this.’ ‘Our clinical systems are quite complex and don’t necessarily facilitate good connected care—we need systems to do that.”
Patient experience of care (subtheme)	Participants believed that care navigation improved the patient experience of care but were unsure if this led to better outcomes, such as reduced hospital admissions.	“As nice as it was for patients for feeling better, the readmission rate wasn’t reduced. The quality of care improved, the quality of discharge improved but in terms of the outcome we were looking at there was no obvious benefit.”

**Table 2:** i-PARIHS framework on targeted care navigation implementation.

i-PARIHS Construct	Theme/Subtheme(s)	Enablers	Barriers
Innovation	Patient experience of care	Perceived improvement in experience and quality of care	Unclear/no benefit for patient outcomes
	Staff and resources	Algorithm helped to identify and target those most at risk.	
	Health service culture	Care navigation model aligns with staff members’ professional values	
Recipients	Patient experience of care	Staff reassured by level of care provided to patients Perception that patients are reassured by level of care received	Belief that some people with chronic disease cannot benefit from the care navigation model
	Staff and resources	Highly skilled clinician	Lack of staff working on the project
Context (inner): local	Leadership	Commitment from managers to trial care navigation model in the future	Unclear key performance indicators
Context (inner): organisational	Staff and resources	Established community health services are sufficient to support patients on discharge from hospital.	Clinical systems are a barrier to coordinating care for people discharging from hospital.
			Limitations of IT systems to “flag” patients at risk of readmission
	Leadership		Lower organisational priority compared to traditional medicalised models of care
			Risk averseness
Context (outer): external health system	Leadership		Opaque communication between government and health service
			Lack of financial incentive to shift care from hospital-based to community-based services
Facilitation	Staff and resources	Skilled clinician helped to facilitate implementation of care navigation model	Inadequate staffing to facilitate the implementation of care navigation

## Data Availability

Data are contained within the article.

## Appendix A

**Table A1:** Semi-structured interview questions and the corresponding Theoretical Domains Framework.

Question	TDF Domain
What is your understanding of discharge support and drivers of hospital readmission?	Knowledge
What was your role in the project? What skillset/previous experience did you bring to the project?	Skills
Do you think the use of readmission risk algorithm best described the patients at risk of hospital readmission? Why?	Social/professional role and identity
Comment on (i) Targeted Care Navigation cohort (ii) Hospital eligibility criteria (iii) services/model of care and (iv) define KPI	N/A
How does this project align with your professional identity and professional standards?	Social/professional role and identity
How difficult or easy was it for you to implement the program?	Beliefs about capabilities
What do you see were the main enablers for implementing this project? Explain	N/A
What do you see were the main barriers for implementing this project? Explain	N/A
How confident were you that it could be implemented?	Optimism
What do you see were the strengths of the project? Explain	N/A
What do you see were the limitations of the project? Explain	N/A
What do you think would happen/or what would be the consequences of permanently implementing this (both positive and negative)?	Beliefs about consequences
Are there any incentives to do this? (personal, program, organisational and Departmental level)	Reinforcement
Have you made a decision whether or not you would like to have this project implemented?	Intentions
How much would you like to see this type of intensive care coordination implemented?	Goals
Can you see this type of intervention being something that you usually do (become part of routine practice)	Memory, attention and decision processes
To what extent do physical or resource factors facilitate or hinder you to do intensive care coordination? e.g., Training; Management meetings	Environmental context and resources
To what extent did peers/managers/patients facilitate or hinder use of the intervention?	Social influences
Does using the package evoke an emotional response? If so, what?	Emotion
Are there procedures or processes can be in place that better encourage intensive care coordination?	Behavioural regulation
How can we make it sustainable?	N/A

N/A: not applicable and/or cannot be mapped to a single TDF domain; TDF: Theoretical Domains Framework.
